# Efficacy of Cyclin Dependent Kinase 4 Inhibitors as Potent Neuroprotective Agents against Insults Relevant to Alzheimer’s Disease

**DOI:** 10.1371/journal.pone.0078842

**Published:** 2013-11-11

**Authors:** Priyankar Sanphui, Sumit Kumar Pramanik, Nandini Chatterjee, Ponnusamy Moorthi, Biswadip Banerji, Subhas Chandra Biswas

**Affiliations:** 1 Cell Biology and Physiology Division, CSIR-Indian Institute of Chemical Biology, Kolkata, India; 2 Chemistry Division, CSIR-Indian Institute of Chemical Biology, Kolkata, India; Case Western Reserve University, United States of America

## Abstract

Alzheimer’s disease (AD) is a progressive neurodegenerative disease with no cure till today. Aberrant activation of cell cycle regulatory proteins is implicated in neurodegenerative diseases including AD. We and others have shown that Cyclin dependent kinase 4 (Cdk4) is activated in AD brain and is required for neuron death. In this study, we tested the efficiency of commercially available Cdk4 specific inhibitors as well as a small library of synthetic molecule inhibitors targeting Cdk4 as neuroprotective agents in cellular models of neuron death. We found that several of these inhibitors significantly protected neuronal cells against death induced by nerve growth factor (NGF) deprivation and oligomeric beta amyloid (Aβ) that are implicated in AD. These neuroprotective agents inhibit specifically Cdk4 kinase activity, loss of mitochondrial integrity, induction of pro-apoptotic protein Bim and caspase3 activation in response to NGF deprivation. The efficacies of commercial and synthesized inhibitors are comparable. The synthesized molecules are either phenanthrene based or naphthalene based and they are synthesized by using Pschorr reaction and Buchwald coupling respectively as one of the key steps. A number of molecules of both kinds block neurodegeneration effectively. Therefore, we propose that Cdk4 inhibition would be a therapeutic choice for ameliorating neurodegeneration in AD and these synthetic Cdk4 inhibitors could lead to development of effective drugs for AD.

## Introduction

Worldwide 36 million people were living with dementia in 2010 and it may increase to 115 million by 2050 (http://www.alz.co.uk/research/files/WorldAlzheimerReport.pdf). Alzheimer’s disease (AD) is most common form of dementia that accounts for 60–80% cases and has no cure. Currently few symptomatic treatments are available that provide mild benefits which are nevertheless dose dependent [1]. Several attempts have been taken for development of disease modifying therapies. These are mostly targeting synthesis or clearance of beta-amyloid (Aβ), which is thought to be central to the disease. Aβ is generated from a trans-membrane protein, amyloid precursor protein (APP) by sequential cleavages with β-, and γ-secretases [2], [3]. Recently, most promising drugs that target either these enzymes by inhibitors or clearing Aβ by immunotherapy have failed in phase 3 clinical trials [4] It raises the question of targeting classical pathways that may govern AD.

The pathological hallmarks of AD are i) extracellular Aβ plaques, ii) intracellular neurofibrillary tangles and iii) extensive neuronal loss due to apoptosis. One of the major causes of neuronal apoptosis is aberrant activation of cell cycle molecules. Differentiated neurons are post-mitotic and stay in G_0_ of cell cycle. However, in AD, accumulating evidence suggests that neurons vulnerable to degeneration emerge from non-dividing state to cycling state with expression/activation of cell cycle markers [5], [6], [7], [8]. However, neurons are unable to complete mitosis due to lack of factors for nuclear division and cytokinesis [9]. Recent studies have indicated a sequential and multi-step pathway of cell cycle that is initiated by various apoptotic insults relevant to AD and that is required for neuron death. The first step in this apoptotic cascade is rapid activation of the G1/S kinase Cdk4. This in turn hyperphosphorylates proteins of the Retinoblastoma (Rb) family, leading to dissociation of a repressor complex comprised of Rb family members and E2 promoter binding factor (E2F) transcription factors. Ultimately, these events lead to induction of a pro-apoptotic gene Bim which in turn activates effectors caspases that lead to demise of neurons [7].

Interestingly, it has been found that cell cycle events in neurons appear in brain of AD patients at very early stages of the disease [10], [11]. Cell cycle re-entry actually occurs prior to development of Aβ plaques and formation of neurofibrillary tangles in many disease models and human patients of AD [5]. Consistent with this, it has been found that forced induction of cell cycle in forebrain of a novel transgenic mouse lead to neuron death, gliosis and cognitive impairment as in AD [12]. Therefore, inhibition of cell cycle re-entry in neurons could be potential therapeutic strategy in AD.

Cyclin D1/Cdk4 activity is required for G1/S transition of cell cycle. A growing number of reports indicate that the kinase activity of Cdk4 is inappropriately increased in neurons in response to various apoptotic stimuli [7], [13], [14]. Moreover, a number of pan-CDK inhibitors protect neurons from death stimuli relevant to AD [15], [16]. More importantly, downregulation of Cdk4 by expression of a dominant or shRNA constructs provide significant protection against various insults implicated in AD [15], [17]. However, in vivo use of these genetic tools has offered off-target effects or other critical limitations. Therefore, specific small molecule inhibitors against Cdk4 might be a better choice for therapeutic purpose.

Altered metabolism of Aβ, particularly accumulation of oligomeric form of Aβ_1–42_ peptide is well accepted underlying cause of pathophysiology of AD [2]. The treatment of neuronal cells with oligomeric Aβ_1–42_ induces death [18], [19], and has been widely used as a good *in vitro* model of the disease. On the other hand, NGF deprivation is a major cause of developmental neuronal pruning and has been implicated in various neurodegenerative diseases including AD [20]. TrkA, the receptor for NGF also shown to be reduced in early-stage AD and this decline is associated with cognitive decline [21]. Accumulating evidences also link lack of NGF signaling to altered amloidogenesis and development of Alzheimer’s pathology [22], [23]. Transgenic mice that express a neutralizing anti-NGF recombinant antibody developed AD pathology including amyloid plaques, neurofibrillary tangles with behavioural deficit [24], [25]. Moreover, an adeno-associated virus based vector expressing human NGF is on clinical trial for the disease [26]. Therefore, neuronal cell death induced by NGF deprivation *in vitro* may represent neurodegeneration seen in AD.

In this study, we employed rat pheochromocytoma (PC12) cells, those are neuroprecursor cells and become differentiated in presence of NGF [27]. We also used primary cultures of rat cortical neurons for this study. We tested two commercially available and ten synthesized small molecule Cdk4 inhibitors for their neuroprotective efficacy in these cells after death insults. We found that Cdk4 specific inhibitors protected neuronally differentiated PC12 cells against NGF deprivation and cortical neurons against Aβ exposure. We also found that Cdk4 inhibitors block the apoptotic cell cycle pathway that is activated in neuronal cells in response to NGF deprivation.

## Results and Discussion

### Synthesis of Small Molecule Inhibitors Against Cdk4

**Figure 1:**
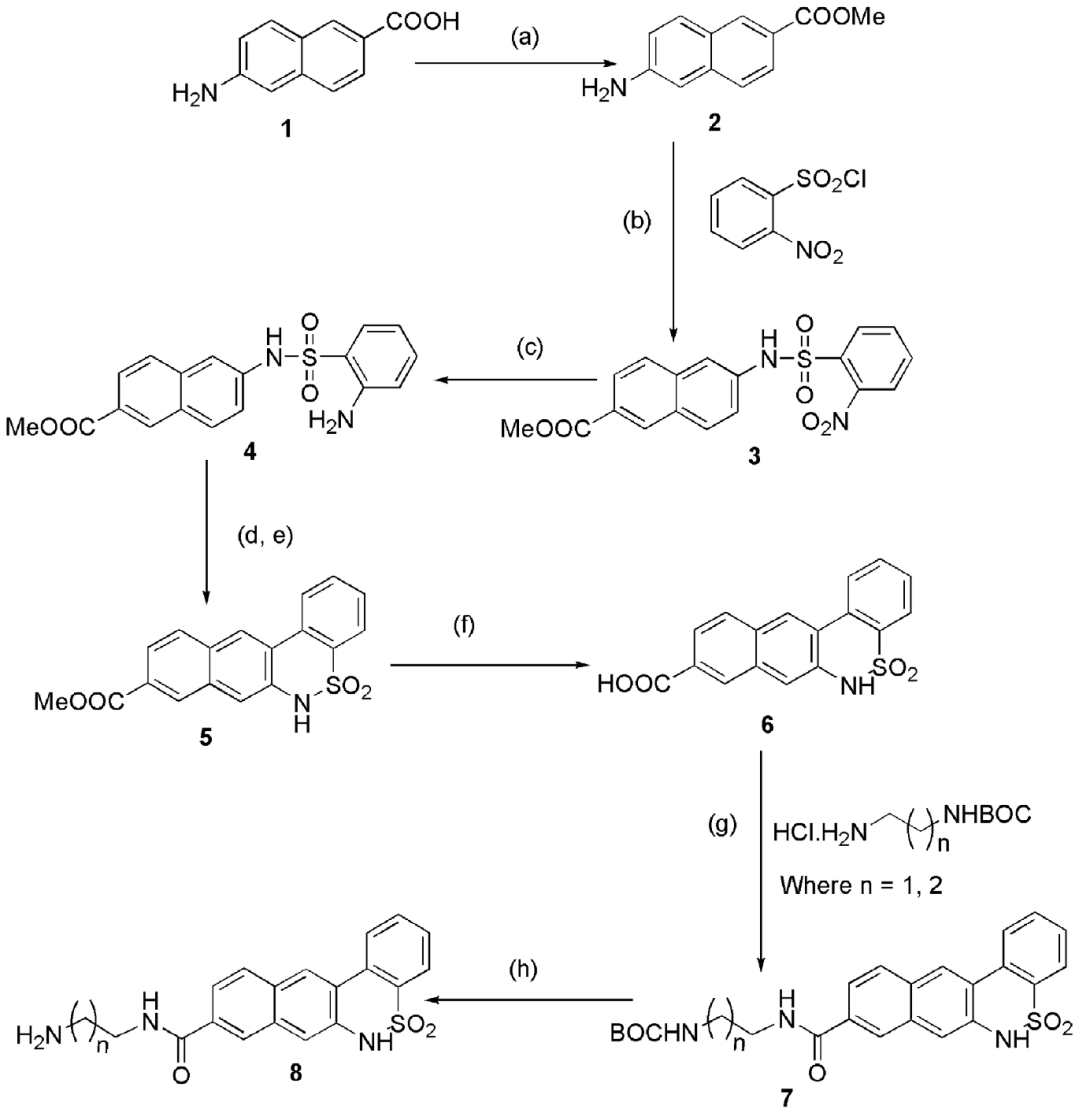
General scheme for synthesis of compound 8. Reagent and conditions: (a) Conc. H_2_SO_4_, MeOH, 0°C to rt, 6 hrs. (b) Triethylamine(1.5 equiv.), THF, 0°C - r.t., 4 hrs. (c) SnCl_2_ (3.0 equiv.), ethanol, reflux, 2 hrs, (d) NaNO_2_(aq), HCl-AcOH, 0°C, 0.5 hrs. (e) Cu powder, 70°C. (f) Lithium hydroxide (1.5 equiv.), MeOH-water (5∶1), 0°C to rt, 1.5 hrs. (g) EDC.HCl (1.5 equiv.), HOBT (1.2 equiv.), TEA (3 equiv.), 0°C to rt, 7.0 hrs. (h) 4(M) HCl in 1,4 dioxane, 0°C to rt, 2.0 hrs.

**Figure 2:**
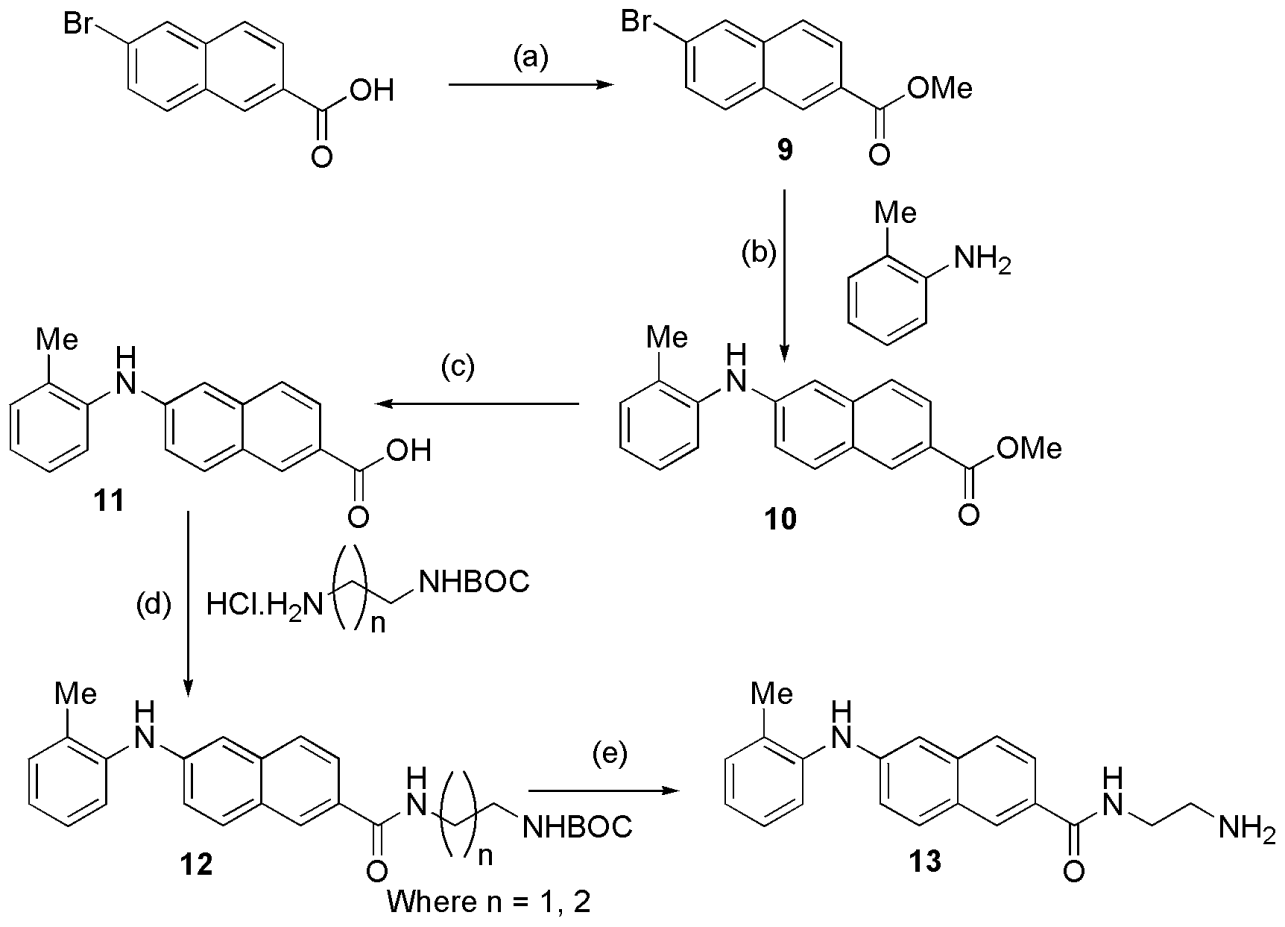
General scheme for synthesis of compound 13. Reagent and conditions: (a) Conc. H_2_SO_4_, MeOH, 0°C to rt, 6 hrs. (b) palladium(II) acetate (0.05 equiv.), xantphos (0.1 equiv.) and cesium carbonate (3 equiv.), 80°C, 4 hrs. (c) Lithium hydroxide (1.5 equiv.), MeOH-water (5∶1), 0°C to rt, 1.5 hrs. (d) EDC.HCl (1.5 equiv.), HOBT (1.2 equiv.), TEA (3 equiv.), 0°C to rt, 7.0 hrs. (e) 4(M) HCl in 1,4 dioxane, 0°C to rt, 2.0 hrs.

**Figure 3:**
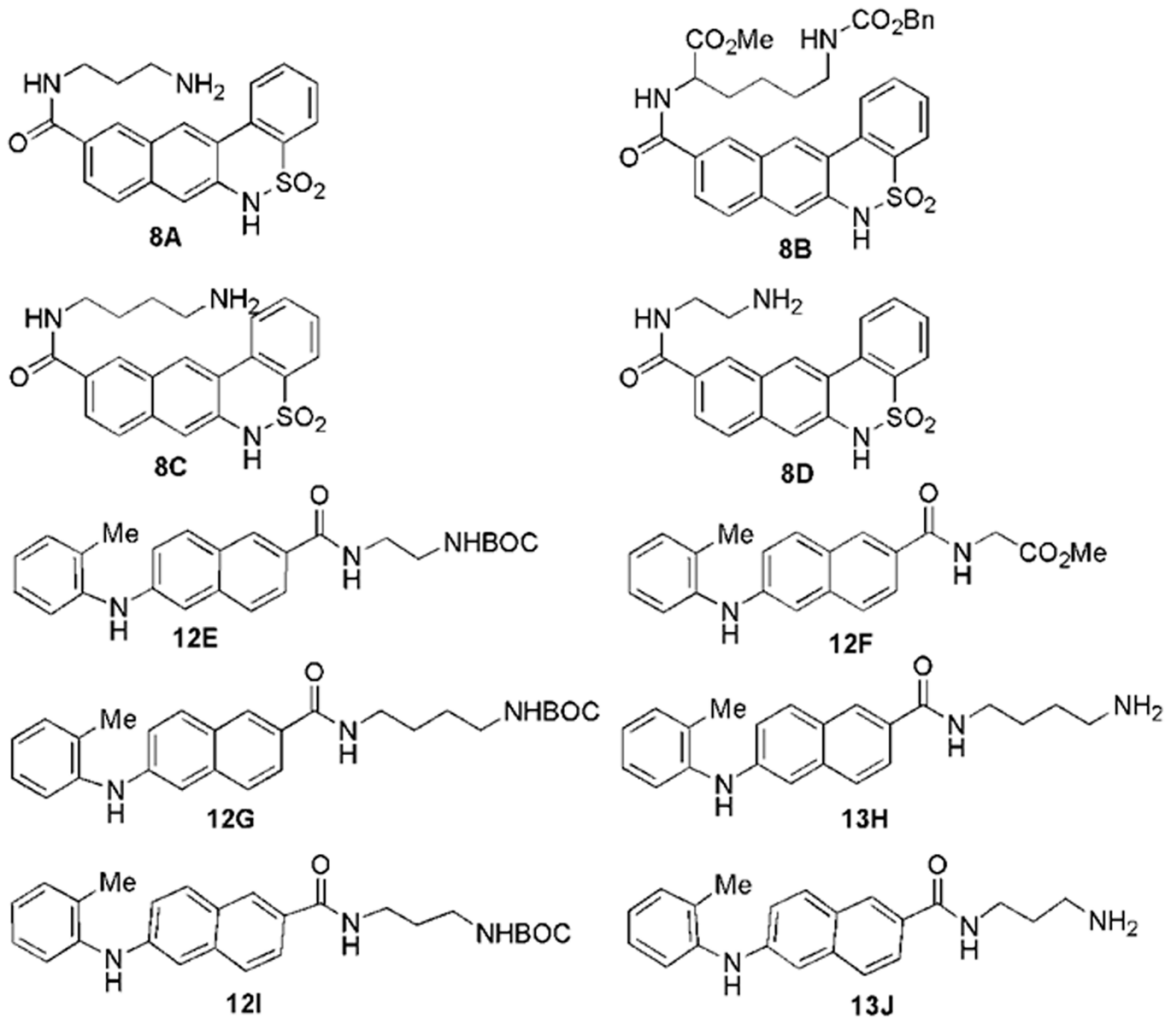
The structures of synthesized Cdk4 inhibitors.

A number of diverse scaffolds were synthesized as Cdk4 inhibitors. The designs of the molecules were guided by the modifications based on already known inhibitors in the series. Pschorr reaction [28] was the key step in case of Figure 1, whereas Buchwald coupling reaction [29] resulted in the synthesis of another set of molecules as shown in Figure 2. All together ten new inhibitors were synthesized according to the scheme as shown in Figure 3.

### Small Molecule Inhibitors of Cdk4 Protect Neuronal Cells Against NGF Deprivation

**Figure 4:**
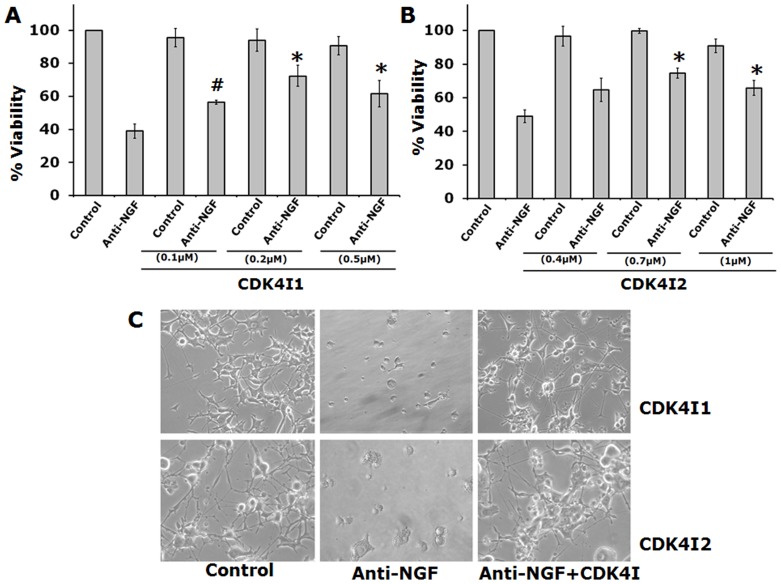
Commercial inhibitors of Cdk4 protect neuronally differentiated PC12 cells against NGF deprivation. Cells were subjected to NGF deprivation in presence and absence of commercially available small molecule inhibitors of Cdk4 for 20(A) graphical representation of percentage of viable cells following NGF deprivation in presence of indicated concentrations of commercially available Cdk4 inhibitor (Cdk4I1). Data represented as mean ± SEM of three independent experiments performed in duplicates. Theasterisks and hash denote statistically significant differences between indicated class:#p<0.05; *p<0.01. (B) Graphical representation of percentage of viable cells following NGF deprivation in presence of indicated concentrations of commercially available Cdk4 inhibitor Cdk4I2. Data represented as mean ± SEM of three independent experiments performed in duplicates. The asterisks denote statistically significant differences between indicated class:*p<0.01. (C) Representative phase contrast micrographs show retention of neuronal processes even after NGF deprivation in presence of commercially available Cdk4 inhibitors (Cdk4I1 & Cdk4I2).

PC12 cells are neuronally differentiated by addition of NGF as described previously [27]. NGF deprivation results in death of PC12 cells and it has been widely used to study mechanism of neuronal cell death [7]. Since NGF deprivation is implicated in AD [21], [22], we employed this cellular model of neuron death for screening of number of Cdk4 inhibitors for their neuroprotective potentials. First two commercially available Cdk4 inhibitors, Cdk4 inhibitor I (Cdk4I1) and Fascaplysin chloride (Cdk4I2) were used. To determine the effective doses, we performed a dose-response curve for each inhibitor depending on the IC_50_ value indicated in the product data sheet. IC_50_ value of Cdk4I1 is 0.076 µM. As shown in Figure 4A, Cdk4I1 showed significant protection of neuronal PC12 cells following NGF deprivation at a concentration of 0.1 µM. This inhibitor provided highest protection (53%) at a concentration of 0.2 µM. However, at higher concentrations it did not provide better protection and showed toxicity to control cells (Figure 4A and unpublished data). IC_50_ value of Cdk4I2 is 0.35 µM. Cdk4I2 showed protection of neuronal PC12 cells from death evoked by NGF deprivation at a concentration of 0.4 µM with highest protection (47%) at a concentration of 0.7 µM (Figure 4B). This compound was also toxic to control cells at higher concentrations (Figure 4B and unpublished data). Importantly, cells maintained in presence of these inhibitors retained the overall neuronal morphology including neuronal processes and network after NGF deprivation (Figure 4C).

**Figure 5:**
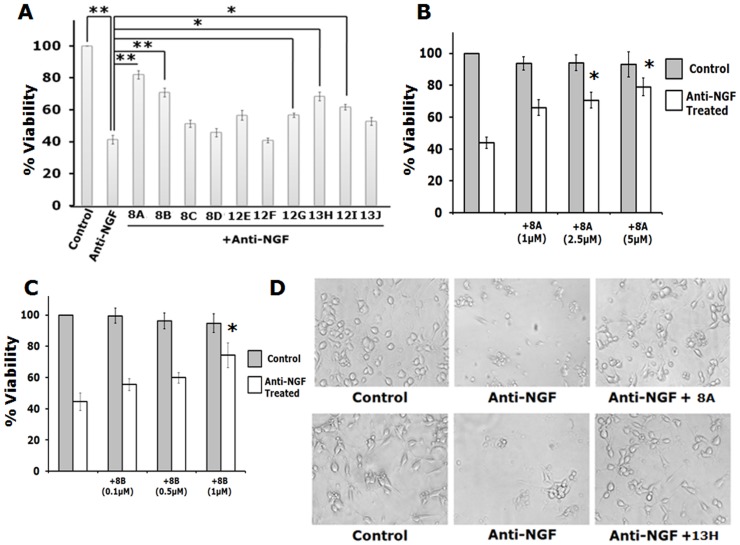
Synthesized inhibitors of Cdk4 protect neuronally differentiated PC12 cells against NGF deprivation. Cells were subjected to NGF deprivation in presence and absence of synthesized small molecule inhibitors of Cdk4 for 20(A) Graphical representation of percentage of viable cells following NGF deprivation in presence of synthesized small molecule Cdk4 inhibitors. Doses of each molecule are 8A: 5 µM; 8B: 1 µM; 8C: 1 µM; 8D: 5 µM; 12E: 1 µM; 12F: 5 µM; 12G: 1 µM; 13H: 1 µM; 12I: 1 µM; 13J: 1 µM. Data represented as mean ± SEM of three independent experiments performed in duplicates. The asterisks denote statistically significant differences between indicated class: **p<0.01, *p<0.05. (B) Dose kinetics of synthesized small molecule inhibitor 8A in differentiated PC12 cells in presence and absence of NGF. Data represented as mean ± SEM of three independent experiments. The asterisks denote statistically significant differences between indicated classes: *p<0.05. (C) Dose kinetics of synthesized small molecule inhibitor 8B in differentiated PC12 cells in presence and absence of NGF. Data represented as mean ± SEM of three independent experiments. The asterisks denote statistically significant differences between indicated classes: *p<0.05. (D) Representative phase contrast micrographs show retention of neuronal processes even after NGF deprivation in presence of synthesized Cdk4 inhibitors (8A & 13H).

Next we screened the synthesized small molecule inhibitors for their biological activity. Different doses of the chemical inhibitors were used for the screening. The doses mentioned were the lowest dose at which the compounds showed protective effect without significant toxicity on control cells. Out of the ten molecules tested, five significantly protected PC12 cells against NGF deprivation (Figure 5A). Of the five molecules that protected cells, two molecules were highly protective and one showed better neuroprotective effect than the commercially available inhibitors tested. We further performed dose response studies of these two compounds, 8A and 8B, and found that they have protected neuronal PC12 cells with increasing concentrations without toxicity to control cells up to 5 µM and 1 µM respectively (Figure 5B and C). The EC50 of 8A and 8B based on their respective dose response curve in this cell death model is 3.18 µM and 0.98 µM respectively. The compounds that protected neuronal PC12 cells also successfully retained the neuronal morphology including neurites and their connections after NGF withdrawal (Figure 5D).

**Figure 6:**
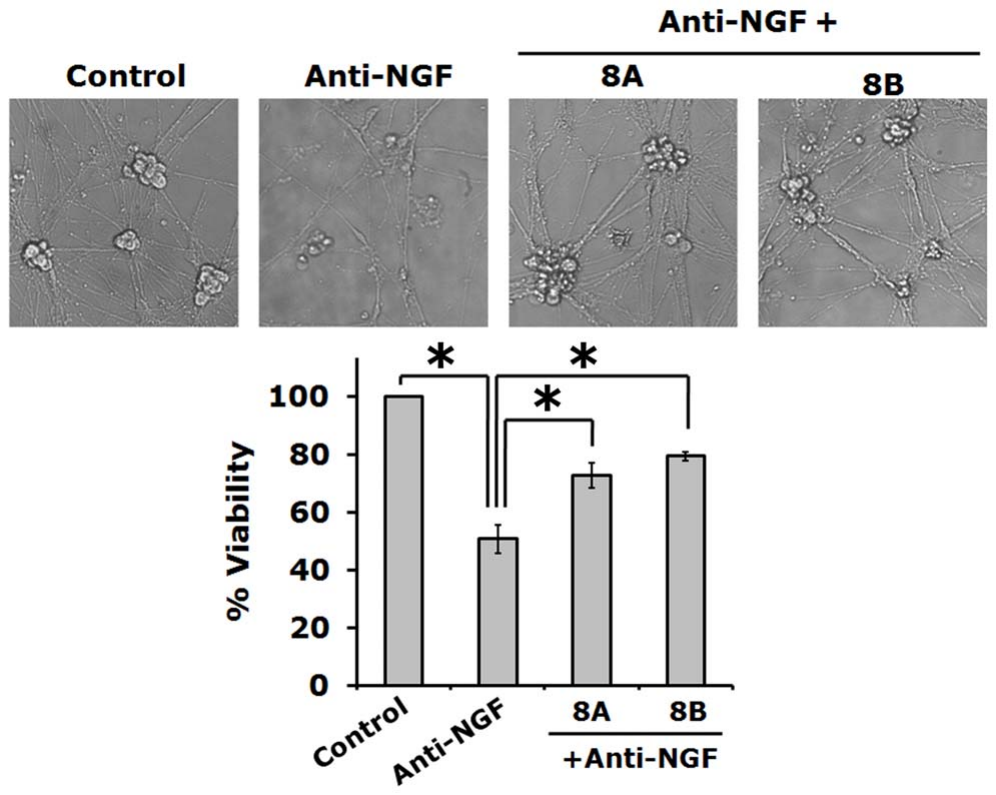
Synthesized inhibitors of Cdk4 protect primary sympathetic neurons against NGF deprivation. Primary cultures of sympathetic neurons (5DIV) were subjected to NGF deprivation in presence and absence of synthesized small molecule inhibitors of Cdk4 for overnight. (A) Representative phase contrast micrographs show retention of neuronal processes even after NGF deprivation in presence of synthesized Cdk4 inhibitors (8A & 8B). (B) Graphical representation of percentage of viable cells following NGF deprivation in presence of synthesized small molecule Cdk4 inhibitors (8A: 5 µM; 8B: 1 µM). Data represented as mean ± SD of two independent experiments performed in duplicates. The asterisks denote statistically significant differences between indicated class: *p<0.05.

Since immortalized cell lines such as PC12 cells may behave differently, we have also used primary cultures of rat sympathetic neurons to test neuroprotective efficacy of synthesized compounds, 8A and 8B. As expected these compounds also protected from neurite loss and death of sympathetic neurons in response to NGF deprivation (Figure 6A and B).

### Cdk4 Inhibitors Protect Primary Rat Cortical Neurons Against Aβ toxicity

**Figure 7:**
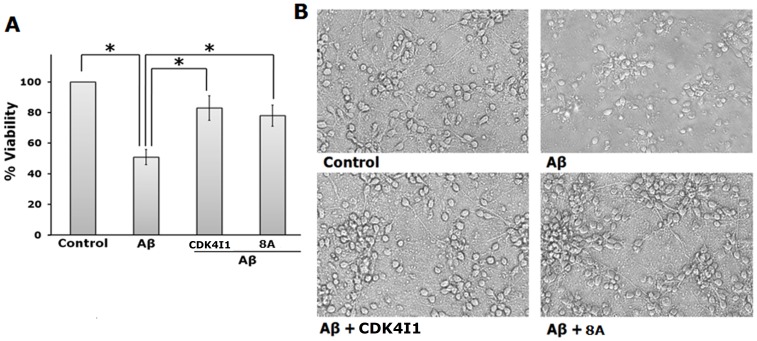
Cdk4 inhibitors protect primary rat cortical neurons against Aβ induced death. Primary rat cortical neurons (5DIV) were exposed to Aβ (1.5 µM) in presence and absence of Cdk4 inhibitors (Cdk5I1 & 8A) for 48 h. Percentage of viable cells were estimated by intact nuclear counting assay. (A) Graphical representation of percentage of viable cells. Data represented as mean ± SD of two independent experiments. The asterisks denote statistically significant differences between indicated class:*p<0.05. (B) Representative phrase contrast micrographs show retention of neuronal processes in presence of Aβ by Cdk4 inhibitors.

Neuron death and degeneration in specific regions of brain are the underlying cause of clinical symptoms of AD [30]. Aβ has been placed central of the pathophysiology of the disease and key reason of neuronal loss [31]. Since cortex is predominantly affected in the disease, and cortical neurons undergo death when exposed to oligomeric Aβ *in vitro* [17], we have employed primary rat cortical neurons as model to determine whether Cdk4 inhibitors can protect neurons against Aβ toxicity. We had selected one commercially (Cdk4I1) and one synthesized (8A) inhibitor for this study. Results showed that not only the inhibitors significantly protect the neurons against Aβ toxicity but also successfully retains the neuronal morphology including neuronal branches (Figure 7A and B). Taken together, our results indicate that inhibition of Cdk4 by specific inhibitors provided significant protection towards neuronal cells (both neuronal cell line and primary neurons) against toxins and withdrawal of trophic support that are highly relevant to AD.

### Cdk4 Inhibitors Specifically Block Cdk4 Activity

To check the specificity of Cdk4 inhibitors, first we performed computational chemistry and molecular modeling using one synthesized inhibitor (8A). The crystal structure of Cdk4 protein obtained from Protein Data Bank (PDB ID: 2W9Z). Structures of the synthesized compounds were drawn in Gauss View followed by geometry optimization in Gaussian 09 with DFT level of theory using B3LYP/6–31+G (d,p) basis set. Auto Dock 4.2 and MGLTools of the Scripps Research Institute were used to perform the docking calculations. AutoDock 4 gives a choice of various methods for doing the conformation search: like Genetic Algorithm (GA), Simulated Annealing, Local Search (LS) and Lamarckian GA-LS combination. In our computer, docking calculations were carried out with AutoDock 4.2. The Cdk4 protein and the ligands (compound 8A) were prepared with MGLTools. The Gasteiger partial charges were added in both the cases. The non-polar hydrogen atoms were merged, and the rotatable bonds were defined. At first the whole Cdk4 protein has been placed in a gridbox centered at (−4.232, 1.952, 1.229) of size (44, 76, 66) grid points with a spacing of 1 Å and the compound 8A has been kept as completely rigid. The torsions were allowed to the long side chains of the amino acid residues in the vicinity of the ligand. Docking simulations were performed using the Lamarckian genetic algorithm (LGA) and the Solis & Wets local search method. The initial position, orientation, and torsions of compound 8A were set randomly. All the rotatable torsions were released during the docking procedure. Each docking experiment was derived from 500 independent runs each of which was set to terminate after a maximum of 50,000,000 energy evaluations. The population size was set to 200. The PyMOL [http://www.pymol.org] molecular viewer and the MGLTools were used to render the output and to calculate the distances between atoms. The lowest energy docked conformations were chosen. The Accessible Surface Area (ASA) of the ligands, protein and the complexes were computed by Mark Gerstein’s calc-surface program on Helix Systems server of NIH (http://helixweb.nih.gov/structbio/) using 1.4 Å probe size. The changes in ASA of the ligands and the protein upon complex formation were calculated.

**Figure 8:**
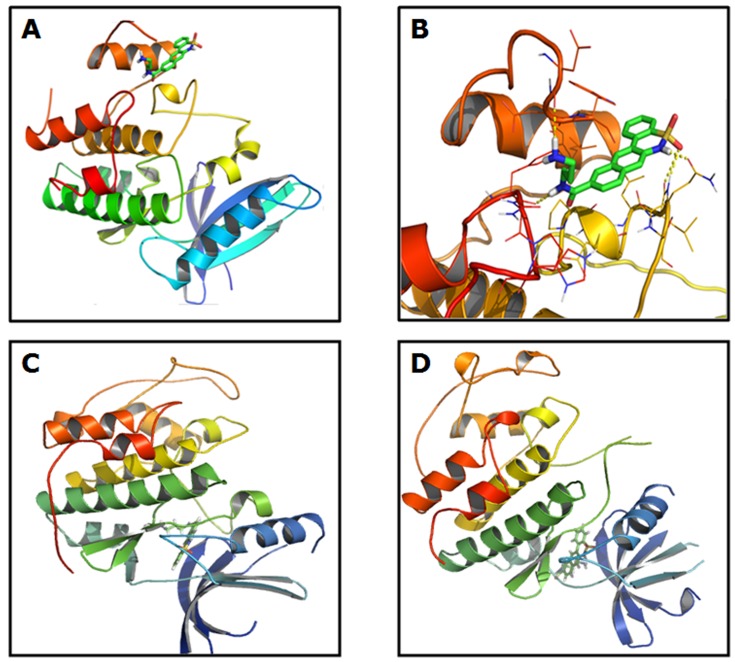
Ribbon representation. (A) Cdk4 docked with best binding modes of compound **8A** along with its close up view. (B). Ribbons are colored in rainbow; blue to red encompassing N-terminal to C-terminal of the proteins. The compound **8A** is shown in stick models colored in the color of respective elements. Here the best binding mode of compound **8A** is found to be at the ATP binding site of Cdk4. (C) Docking of Cdk2 with compound 8A. (D) Docking of Cdk5 with compound 8A.

Docking of a ligand into a protein binding site and estimating the binding affinity of the resulted complex allow understanding the interaction pattern of a small molecule at the binding site. This information provides vital clues to design structure-based drug molecules. Docking analysis in the current investigation carried out to theoretically evaluate the ability of the compounds to bind serum albumins and the binding site of the receptor (Figure 8A). Here the ribbon representation of Cdk4 protein is folded into the typical bilobal structure, with the smaller N-terminal (blue end) domain consisting predominantly of the β-sheet structure and the larger C-terminal (red end) domain consisting predominantly of α-helixes. Here the inhibitor (8A) binds as seen for ATP-Cdk4 complex, i.e. at the ATP binding site. AutoDock study indicates that three residues Arg-62, Glu-43 and Phe-69 in the active site are involved in the binding with the compound 8A through hydrogen bonding. The hydrogen bonding pattern and hydrophobic interactions of compound 8A are shown in figure 8B. From the close-up view it is observed that a total of five hydrogen bonds are present between compound 8A and the Cdk4 protein. These are present between the hydrogen’s of –NH_2_ and the backbone carbonyl of Arg-62; and between the proton of –NH (sulfonamide) and the backbone carbonyl of Glu-43; and between the oxygen of –SO_2_ and backbone proton of NH of Glu-43; and between the proton of amide –NH and the backbone carbonyl of Phe-69. Data indicate that the hydrophobic interactions, Van der Waals attraction and hydrogen bond formation are the major contributing factors in this binding. Rb is found to interact with all the three binding site residues of Cdk4. A strong hydrogen bond is formed between three amino acids Arg-62, Glu-43 and Phe-69 of Cdk4 protein and compound 8A. The binding free energies and the change in the accessible surface area (ASA) of the compound are –30 KJ mol^−1^ and 73.12% respectively.

We have confirmed whether the molecule 8A binds specifically to the Cdk4 at the ATP binding site or to the other Cdks also. For this purpose we have performed the same ‘fitting’ by using Cdk2 and Cdk5 instead of Cdk4 to build the model. The crystal structure of Cdk2 and Cdk5 obtained from Protein Data Bank (PDB ID: 2C6O and 4AU8 respectively). The molecular docking studies were performed in these cases by the same protocol. But in cases of Cdk2 or Cdk5, the compound 8A binds at different binding site than that of Cdk4 (Figure 8C and D). This indicates that this compound is specific to the Cdk4.

**Figure 9:**
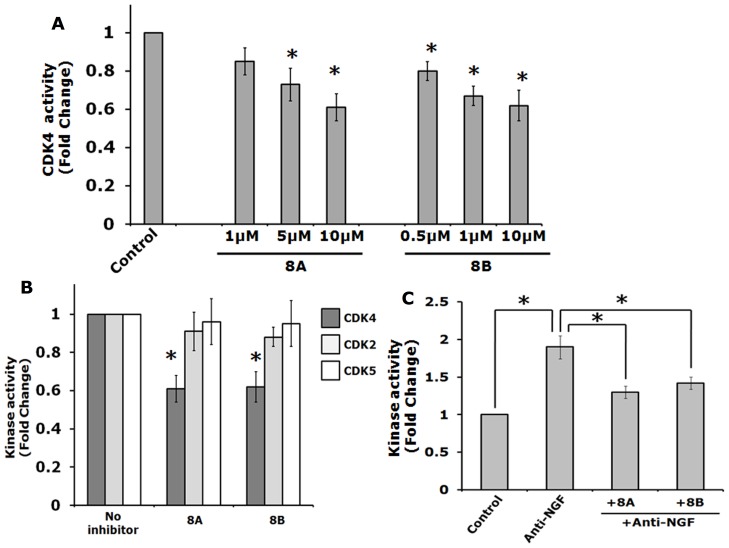
Specificity of Cdk4 inhibitors. (A) Kinase assay of Cdk4 (10 ng) was performed with Cdk4 inhibitors 8A and 8B at different doses as indicated. Data represented as mean ± SEM of 3 independent experiments. The asterisks denote statistically significant differences between indicated classes:*p<0.05. (B) Kinase assay was performed with Cdk4 inhibitors 8A and 8B (10 µM each) with Cdk4, Cdk2 and Cdk5 (10 ng each). Data represented as mean ± STDEV of 2 independent experiments performed in duplicate. The asterisks denote statistically significant differences between indicated classes:*p<0.05. (C) Differentiated PC12 cells were subjected to NGF deprivation in presence or absence inhibitors (8A, 5 µM and 8B, 1 µM) for 8 h. The cells were lysed and equal amount of protein from cell lysate were subjected to immunoprecipitation with Cdk4 antibody. The immunoprecipitated protein was then subjected to kinase assay. Data represented as mean ± SEM of 3 independent experiments. The asterisks denote statistically significant differences between indicated classes:*p<0.05.

To test this computational prediction, next we performed Kinase assays with Cdk4, Cdk2 and Cdk5 in presence and absence of the inhibitors 8A and 8B. First, we performed Cdk4 kinase assay using different doses of these two compounds, 8A and 8B to see the inhibitory potentials of these compounds on Cdk4 activity. We found that these compounds, 8A and 8B significantly blocked kinase activity of Cdk4 at the concentrations that provided neuroprotective effects i.e 5 µM and 1 µM respectively (Figure 9A). Moreover, kinase assays with other Cdks such as Cdk2 and Cdk5 indicated that these inhibitors specifically inhibited Cdk4 and did not block the kinase activity of Cdk2 and Cdk5 (Figure 9B).

We have also checked the endogenous Cdk4 kinase activity after NGF deprivation in presence and absence of the inhibitors 8A and 8B. We immunoprecipitated Cdk4 from total cell lysates of treated and untreated differentiated PC12 cells, then the immunoprecipitated protein was subjected to kinase assay. Results showed about two-fold increase in Cdk4 kinase activity following NGF deprivation in neuronal PC12 cells and that activity was almost completely blocked in presence of the inhibitors (Figure 9C). Taken together, our results suggest that these inhibitors are specific to Cdk4 and they are capable of blocking the NGF deprivation-induced increase of Cdk4 activity.

### Rb Protein Phosphorylation After NGF Deprivation is Blocked in Presence of Cdk4 Inhibitors

**Figure 10:**
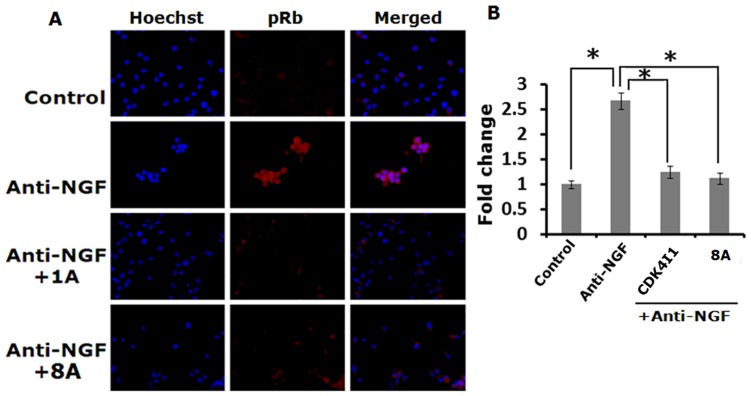
Cdk4 inhibitors block phosphorylation of Rb protein in response to NGF deprivation in neuronal PC12 cells. Cells were subjected to NGF deprivation in presence and absence of Cdk4 inhibitors for 20(A) Representative images show that Cdk4 inhibitors (Cdk4I1 & 8A) blocks NGF deprivation induced elevation of phospho-Rb level. (B) Quantification of the immunostained cells. Intensity of 10–15 cells from each field of 5 random fields was quantified using NIH ImageJ. Data represented as mean ± SEM of 3 independent experiments.

Rb proteins directly bind with E2F proteins and actively repress expression of E2F responsive genes in live neurons. Upon phosphorylation by Cdk4 it translocates out from nucleus to cytosol in response to certain apoptotic stimuli [32]. Phosphorylation of Rb proteins results in dissociation of E2F-Rb repressor complex on E2F responsive pro-apoptotic genes thereby induces expression of those genes [33]. In response to NGF deprivation, Rb proteins are phosphorylated due to activation of Cdk4 [32]. We determined the phosphorylation levels of Rb protein in neuronal PC12 cells after NGF deprivation in presence or absence of Cdk4 inhibitors. Immunocytochemical staining followed by fluorescence imaging studies reveal that intensity of the phospho-Rb staining which is mostly present in cytosol is greatly increased after NGF deprivation and that level is significantly reduced in presence of two Cdk4 inhibitors (one commercial and one synthesized) (Figure 10A and B). These results confirm that these inhibitors render their neuroprotective ability by inhibiting the kinase activity of Cdk4.

### Cdk4 Inhibitors Block Induction of Bim and Activation of Caspase3 by NGF Deprivation

**Figure 11:**
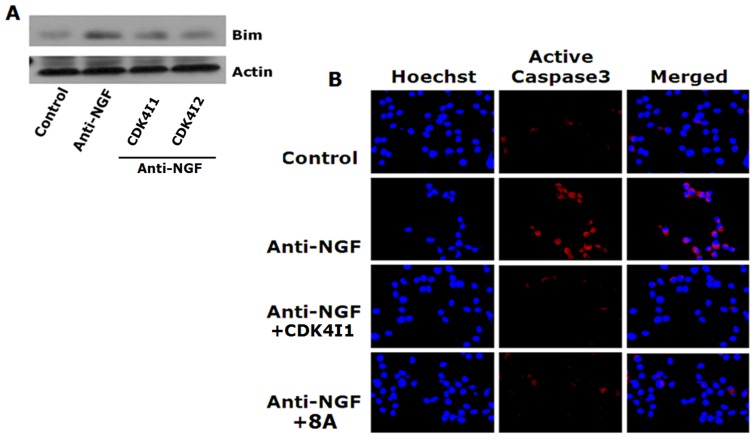
Cdk4 inhibitors block elevation of Bim and active caspase3 levels in response to NGF deprivation in neuronal PC12 cells. Cells were subjected to NGF deprivation in presence and absence of Cdk4 inhibitors for 20(A) Representative immunoblot showing reduction in NGF deprivation induced Bim level by Cdk4 inhibitors (Cdk4I1 & Cdk4I2). (B) Representative images of immunostained cells. Result shows Cdk4 inhibitors (Cdk4I1 & 8A) effectively blocks NGF deprivation induced elevation of active caspase3 level.

Next, we determined the effect of Cdk4 inhibitors on downstream effectors of apoptotic cell cycle pathway that are required for execution of neuron death. Bim is an important pro-apoptotic protein which is induced in neurons and plays a necessary role in neuron death following NGF deprivation or Aβ treatment [17], [34]. We have shown before that expression of Bim is regulated by E2F responsive genes such as mybs those are induced through Cdk4 mediated Rb phosphorylation in neurons after NGF deprivation or Aβ treatment [17], [35]. So we intended to test whether the Cdk4 inhibitors can block the NGF deprivation induced upregulation of Bim. Western blotting analysis showed that a pronounced reduction of NGF deprivation induced upregulation of Bim by Cdk4 inhibitors. Both inhibitors used were equally effective in inhibiting Bim induction upon NGF deprivation (Figure 11A).

Caspase3 is the major effectors of apoptosis which is activated by Bim and other similar proteins in response to apoptotic stimuli [36]. Active caspase3 has also been shown to be required for synaptic dysfunction as well as neuronal loss in AD [36], [37]. So we next checked the level of active caspase3 in neuronal PC12 cells following NGF deprivation in presence or absence of Cdk4 inhibitors. For this purpose we selected one commercial (Cdk4I1) and one synthesized inhibitor (8A). Immunocytochemical staining followed by fluorescence imaging showed that, the inhibitors successfully blocked the NGF deprivation induced elevation of active caspase3 (Figure 11B). Collectively, these data suggest that Cdk4 inhibitors block the apoptotic cell cycle pathway in neuronal cells that is activated and executes neuron death in response to NGF deprivation.

## Conclusions

Currently no disease modifying therapy is available to treat patients of AD. Recent research elucidated the pathophysiology of the disease. Pathologically the disease is characterized by senile plaques composed of Aβ peptides, neurofibrillary tangles composed of paired helical filaments of phosphorylated tau protein and neurodegeneration due to multiple causes such as aberrant activation of cell cycle, oxidative stress, neuroinflammation etc. [7], [30]. However, it has been proposed that the primary cause of AD is accumulation of Aβ in the brain which is known as ‘amyloid cascade hypothesis’ [3]. According to this hypothesis, the rest of the disease process including formation of neurofibrillary tangles and neurodegeneration is proposed to result from imbalance between Aβ formation and clearance [3]. Accordingly, during the last decade number of drugs targeting Aβ formation such as inhibitors of its processing enzymes β- and γ-secretases, and Aβ clearance such as immunotherapy using active or passive antibodies against Aβ were in clinical trials and were very promising. Very recently, most of them have been failed in phase 3 clinical trials [4]. These failures raise the question of targeting Aβ metabolism for ameliorating neurodegeneration in AD and suggest testing other avenues for the purpose.

Aberrant activation of cell cycle markers in vulnerable neurons in AD brain has been reported by several groups [6]–[8], [38], [39] and that occur prior to the development of neurofibrillary tangles and Aβ plaques [40]. Cell cycle proteins are also activated in cellular and animal models of the disease and inhibition of cell cycle re-entry blocks neurodegeneration in these models [7], [32]. More specifically, G1/S transition kinase Cdk4 activity is enhanced in vulnerable neurons in AD brains and inhibition of its activity by d/n or siRNA constructs protected neurons from death induced by NGF deprivation or Aβ exposure [15]–[17], [35]. These genetic tools have critical limitations such as off-target effects and further application as therapeutic agents. Specific small molecule inhibitors are suitable for *in vivo* use and further development as drugs. In this study we have tested specific Cdk4 inhibitors in cellular models of neuron death relevant to AD and found their protective effects in neurons that are affected in AD. We have used two commercial and ten novel molecules synthesized by expedited methods. Two commercial and five synthesized molecules provided significant protection of neuronal cells against trophic support deprivation. Their neuroprotective efficacy also tested in primary cortical neurons those were exposed to oligomeric Aβ which has been thought to be primary cause of AD pathogenesis. Moreover, these inhibitors not only protected neuronal cell body but also neuronal processes and connections those are lost in response to Aβ. Most importantly, they are not toxic to normal cells and effective in low doses. Therefore, these novel synthesized molecules may lead to development of effective drugs to ameliorate neurodegeneration in AD.

It has been reported that activation of Cdk4 by certain apoptotic stimuli leads to phosphorylation of Rb proteins and subsequent expression of E2F responsive pro-apoptotic genes. Expression of E2F-responsive genes such as transcription factors B- and C-myb cause induction of a pro-apoptotic gene Bim which in turn activates effector caspases and results in neuron death [41]. We found that both commercial and synthesized Cdk4 inhibitors block Rb phosphorylation in response to NGF deprivation, means they effectively block kinase activity of Cdk4. Moreover, these synthesized molecules specifically block kinase activity of Cdk4, but not Cdk2 or Cdk5. These inhibitors also block Bim induction and activation of effector caspase3 in neuronal cells after NGF deprivation. Taken together, our results strongly indicate that Cdk4 inhibition might provide effective neuroprotection in AD and our newly synthesized small molecule inhibitors may lead to development of new drugs against neurodegeneration in AD.

## Materials and Methods

### Materials

Cdk4 inhibitor, 2-Bromo-12,13-dihydro-5H-indolo [2,3-a]pyrrolo [3,4-c]cabazole-5,7(6H)-dione (Cdk4I1) was purchased from Calbiochem (La Jolla, CA) and Fascaplysin chloride, 12,13-Dihydro-13-oxopyrido [1,2-a:3,4-b’]diindol-5-ium chloride (Cdk4I2) was purchased from sigma (St. Louis, MO, USA). All cell culture media, sera and Alexa Fluor568 were purchased from Invitrogen (Life technologies, Grand Island, NY, USA). Human recombinant NGF, insulin, progesterone, putrescine, selenium, transferrin and poly-D-lysine were purchased from Sigma (St. Louis, MO, USA). Anti-Bim antibody was from Abcam (Cambridge, UK), Anti-phospho Rb antibody was from cell signaling (Denver, MA, USA), anti-Cdk4 antibody, Protein A agarose and HRP-conjugated secondary antibodies were from Santa Cruz Biotechnology (Dallas, Texas, USA). Cdk4, Cdk2 and Cdk5 were purchased from SignalChem (Richmond, BC, Canada).

### Chemistry

All air and water sensitive reactions were carried out in oven dried glassware under nitrogen atmosphere using standard manifold techniques. All the chemicals were purchased from across organics and sigma-Aldrich, and used without further purification unless otherwise stated. Compounds that are not described in the experimental part were synthesized according to the literature procedures. Solvents were freshly distilled by standard procedures prior to use. Flash chromatography was performed on silica gel (Merk, 100–200 mesh) with the indicated eluant. All ^1^H and ^13^C-NMR spectra were recorded on a Bruker 600 MHz spectrometer. For ^1^H NMR, tetramethylsilane (TMS) served as internal standard (δ = 0) and data are reported as follows: chemical shift, integration, multiplicity (s = singlet, d = doublet, t = triplet, q = quartet, m = multiplet) and coupling constant(s) in Hz. For ^13^C NMR, TMS (δ = 0) or CDCl_3_ (δ = 77.26) was used as internal standard and spectra were obtained with complete proton decoupling. Mass spectra were obtained on a Jeol MS station 700 and ESI-TOF mass spectrometer. FT-IR Spectra were recorded on a JASCO FT/IR−4200 spectrometer.

### General Procedure to Synthesize Compound 8

To a stirred solution of 6-Amino-naphthalene-2-carboxylic acid (compound **1,** Figure 1) (1.0 equiv.) in methanol was added H_2_SO_4_ (10% by mass) at 0°C. The reaction was then refluxed for 6 hours. The reaction mixture was then neutralized by 1 M NaOH solution and extracted with ethyl acetate. Then the ethylacetate portion was washed with water and dried over sodium sulphate and then concentrated to dryness. After that the residue was purified by column chromatography (hexane/ethyl acetate) to afford 6-Amino-naphthalene-2-carboxylic acid methyl ester (compound **2,** Figure 1).

To a stirred solution of compound **2** (1.0 equiv.) in tetrahydrofuran was added 2-nitrobenzenesulfonyl chloride (1.0 equiv.) and triethylamine (1.2 equiv.) at 0°C. The reaction was stirred at room temperature for 4 hours. After this period the reaction mixture was evaporetad to dryness, diluted with EtOAC and water. The ethylacetate portion was extracted and dried over sodium sulphate. Then this ethyl acetate was concentrated to dryness, and the residue was purified by column chromatography (hexane/ethyl acetate) to afford compound **3 (** Figure 1).

To a stirred solution of compound **3** (1.0 equiv.) in ethanol was added stannous chloride (3.0 equiv.) and then the reaction mixture was refluxed for 2 hours. Ethanol was then evaporated under reduced pressure and the reaction mixture was then neutralized with 1 M NaOH solution and extracted with ethyl acetate. The ethyl acetate layer was dried over sodium sulphate and concentrated to dryness. Then the residue was purified by column chromatography (hexane/ethyl acetate) to afford compound **4 (** Figure 1).

To a stirred solution of compound **4** (1.0 equiv.) at 0°C in acetic acid and HCl (3∶1) mixture was added aq. NaNO_2_ and the mixture was stirred for 0.5 hrs at the same temperature. After this period copper powder was added and stirred vigorously for 40 minutes at 70°C. The reaction mixture was then neutralized by 1 M NaOH solution and extracted with ethyl acetate. The ethyl acetate layer was concentrated to dryness, and the residue was purified by column chromatography (hexane/ethyl acetate) to afford compound **5 (** Figure 1).

To a stirred solution of compound **5** (1 equiv.) in MeOH-water (5∶1) was added lithium hydroxide (1.5 equiv.) at 0°C. The reaction was then continued at room temperature for 1.5 hours. MeOH was then evaporated and the solution was then neutralized by using 1 (M) HCl solution and the compound was then extracted with ethyl acetate. The ethyl acetate was then concentrated to dryness to afford compound **6 (** Figure 1), which was carried out to the next step without further purification.

To a stirred solution of compound **6** (1 equiv.) and HCl salt of monoboc protected diamine (1.2 equiv.) in dry THF was added EDC.HCl (1.5 equiv.), HOBT (1.2 equiv.) and triethyl amine (3 equiv.) at 0°C. The reaction was then continued at room temperature for 7 hours. The reaction mixture was concentrated to dryness, and the residue was purified by column chromatography (hexane/ethyl acetate) to afford compound **7 (** Figure 1).

To a stirred solution of compound **7** (1.0 equiv.) was added 4 (M) HCl in 1,4-dioxane (5.0 equiv.) at 0°C. The reaction was then continued at room temperature for 4 hours. First the reaction mixture was evaporated to dryness and worked up with ethyl acetate and water. The ethyl acetate was concentrated to dryness, and the residue was purified by column chromatography (hexane/ethyl acetate) to afford compound **8 (** Figure 1).

### General Procedure to Synthesize Compound 13

To a stirred solution of 6-bromo-2-napthoic acid (1 equiv.) in methanol was added H_2_SO_4_ (10% by mass) at 0°C. The reaction was then refluxed for 6 hours. The reaction mixture was then neutralized by 1 M NaOH solution and extracted with ethyl acetate. The ethyl acetate was then concentrated to dryness, and the residue was purified by column chromatography (hexane/ethyl acetate) to afford compound **9 (** Figure 2).

To a stirred solution of compound **9** (1 equiv.) and o-Tolylamine (1.2 equiv.) in 1,4-dioxane was added palladium(II) acetate (0.05 equiv.), xantphos (0.1 equiv.) and cesium carbonate (3 equiv.). The reaction was then continued at 80°C for 4 hours. The reaction mixture was filtered, concentrated to dryness, and the residue was purified by column chromatography (hexane/ethyl acetate) to afford compound **10 (** Figure 2).

To a stirred solution of compound **10** (1 equiv.) in MeOH-water (5∶1) mixture was added lithium hydroxide (1.5 equiv.) at 0°C. The reaction was then continued at room temperature for 1.5 hours. MeOH was then evaporated and the solution was then neutralized by using 1 (M) HCl solution and the compound was then extracted with ethyl acetate. The ethyl acetate was then concentrated to dryness to afford compound **11 (** Figure 2) which was carried out to the next step without further purification.

To a stirred solution of compound **11** (1 equiv.) and HCl salt of monoboc protected diamine (1.2 equiv.) in dry THF was added EDC.HCl (1.5 equiv.), HOBT (1.2 equiv.) and triethyl amine (3 equiv.) at 0°C. The reaction was then continued at room temperature for 7 hours. The reaction mixture was concentrated to dryness, and the residue was purified by column chromatography (hexane/ethyl acetate) to afford compound **12 (** Figure 2).

To a stirred solution of compound **12** (1.0 equiv.) was added 4 (M) HCl in 1,4-dioxane (5.0 equiv.) at 0°C. The reaction was then continued at room temperature for 4 hours. The reaction mixture was worked up with ethyl acetate and water. The ethyl acetate was concentrated to dryness, and the residue was purified by column chromatography (hexane/ethyl acetate) to afford compound **13) (** Figure 2).

### Animals and Ethics

All animal experiments were carried out in accordance with the guidelines formulated by the Committee for the Purpose of Control and Supervision of Experiments on Animals (Animal Welfare Divisions, Ministry of Environment and Forests, Govt. of India), with approval from the Animal Ethics Committee of Indian Institute of Chemical Biology (IICB-AEC). Adult Sprague-Dawley rats, procured from the random bred colony of the animal house of our institute, were maintained under good husbandry conditions supported by diurnal cycles of 12 h light and 12 h darkness with lights on at 06.00 h daily.

### Assessment of Cell Survival

Trypan blue exclusion assay- Trypan blue exclusion assay was performed as described previously [42]. This assay is based on the capability of the viable cells to exclude the dye. As live PC12 cells maintain membrane integrity, the cells do not allow the trypan blue dye to pass through the cell membrane. However, the dying cells that lose their membrane integrity allow the dye to accumulate and appear blue. Neuronally differentiated PC12 cells (5DIV) were maintained in presence and absence of the inhibitors for 24 h. Following NGF deprivation cells were dislodged and cell suspension were mixed with 0.4% trypan blue dye in 1∶1 ratio and then incubated at room temperature for 5 min. Cells were counted in a haemocytometer for the dye uptake. The number of viable cells was calculated as the percentage of total cell population.

Intact nuclei counting assay- This assay was performed as described previously [43]. Neuronally differentiated PC12 cells (5DIV) were maintained in presence and absence of the inhibitors for 24 h after NGF withdrawal. A detergent containing buffer was then added to the cells that dissolve only the cell membrane leaving the nuclear membrane intact. The intact nuclei were then counted on a haemocytometer. The number of live cells was expressed as percentage of the total cell population.

### Cell Culture

Rat Pheochromocytoma (PC12) cells were cultured as described previously [27] in DMEM supplemented with 10% heat-inactivated horse serum and 5% heat-inactivated fetal bovine serum. Neuronal differentiation was induced by DMEM supplemented with NGF (50 ng/ml) and 1% heat-inactivated horse serum (HS). The cells were differentiated for 6 days before performing experiments. Primary Sympathetic neurons were cultured from superior cervical ganglia of newborn rat pups as described previously [44]. Briefly, cells were isolated form superior cervical ganglia and plated on collagen coated 24-well plates in RPMI 1640 medium supplemented with 1% horse serum, Nerve Growth Factor (100 ng/ml), and 2.5 mM each of Uridine and 5-Fluoro-5′deoxyuridine for 5 DIV before treatment. Embryonic rat cortical neurons were cultured as previously described [44]. Briefly, the cortices were dissected out from brains of rat embryo of day 18 and neurons were plated into 24-well dishes (∼500,000 cells/well) coated with poly-D-lysine in serum-free medium (DMEM/F12 [1∶1] supplemented with 6 mg/ml d-glucose, 100 µg/ml transferrin, 25 µg/ml insulin, 20 nM progesterone, 60 µM putrescine, 30 nM selenium). 6 d after plating, the neurons were treated with oligomeric beta-amyloid (Aß).

### Preparation of Aß

HPLC-purified Aß_1–42_ was purchased from American Peptide (Sunnyvale, CA) and oligomeric Aß_1–42_ was prepared as described previously [45]. Briefly, lyophilized Aß_1–42_ was reconstituted in 100% 1,1,1,3,3,3 hexafluoro-2-propanol (HFIP) to 1 mM, HFIP was removed by evaporation in a Speed Vac, then resuspended to 5 mM in anhydrous DMSO. This stock was then stored in −80°C. The stock was diluted with PBS to a final concentration of 400 µM and SDS was added to a final concentration of 0.2%. The resulting solution was incubated at 37°C for 18–24 h. Again the preparation was diluted with PBS to a final concentration of 100 µM and incubated at 37°C for 18–24 h before use.

### NGF Deprivation

Differentiated PC12 cells (5DIV) were washed twice with PBS and feed with fresh DMEM medium containing Anti-NGF.

### Western Blotting

Cells were lysed and protein were analysed by Western blotting as described previously [46]. In short, equal amount of proteins (50 µg) from each condition was resolved by 10% SDS-PAGE. Proteins were transferred on to PVDF membrane (Hybond: GE Healthcare) and probed with anti-Bim antibody (Abcam) for overnight at 4°C on shaker. HRP conjugated secondary antibody against the primary antibodies were used. Amersham ECL Western Blotting detection reagent was used for detection. Bands were detected on X-ray film (Kodak).

### Immunocytochemical Staining

The neuronal cells were immunostained as described previously [17], [35]. In brief, the cells were fixed with 4%PFA for 10 minutes at room temperature. After 3 washes of PBS, the cells were blocked in 3% goat serum in PBS containing 0.1% triton-X 100 for 2 hours. The cells were immuno-labelled with anti-active caspase 3 antibody in blocking solution for overnight at 4°C. Alexa fluor 568 was used as secondary antibody and nuclei were stained with Hoechst.

### Immunoprecipitation

Differentiated PC12 cells were treated with Anti-NGF in presence and absence of inhibitors for 8 h before harvesting. The cells were lysed and the total cell lysates were subjected to immunoprecipitation as described earlier [47]. In brief, the agarose conjugated Cdk4 antibodies were prepared by incubating Cdk4 antibody with Agarose A for 2 h at 4°C in shaking condition. The conjugated antibody was then incubated with the cell lysates containing equal amount of total protein at 4°C overnight under shaking condition. The agarose conjugated antibody bound with the protein was precipitated by centrifugation. The pellets were subjected to kinase assay.

### Kinase Assay

Kinase assays were performed using ADP-Glo Kinase Assay kit from Promega (Madison,WI, USA) following the manufacturer’s protocol. The ADP-Glo Kinase Assay is a luminescent assay that detects the amount of ADP produced in a kinase reaction. Principle of this assay is that ADP generated in the kinase assay is converted to ATP which is then converted to light by Ultra-Glo Luciferase and the light is measured using a luminometer. This kit can be used to measure the activity of any kinase. Briefly, the assay was done in three steps. First, the kinase reaction was done in presence and absence of the inhibitor. Second, the ADP-Glo reagent was added to terminate the reaction and deplete the remaining ATP. In the third step, Kinase Detection Reagent was added which converts the ADP produced in the kinase reaction to ATP. This newly converted ATP is measured using a luciferase/luciferin reaction. The kinase activities of Cdk4, Cdk2 and Cdk5 were measured by using ADP-Glo kinase assay kit. The concentrations used for kinases were 10 ng per reaction and concentrations used for inhibitors were 10 µM per reaction or as indicated in figure.

### Statistics

The experimental results were reported as mean ± SEM. Student’s t-test was performed to evaluate the significance of difference between the means and was presented as p values.
